# Comparison of Electrocardiographic Criteria for Identifying Left Ventricular Hypertrophy in Athletes from Different Sports Modalities

**DOI:** 10.6061/clinics/2017(06)03

**Published:** 2017-06

**Authors:** Nelson Samesima, Luciene Ferreira Azevedo, Luciana Diniz Nagem Janot De Matos, Leandro Santini Echenique, Carlos Eduardo Negrao, Carlos Alberto Pastore

**Affiliations:** I Unidade Clínica de Eletrocardiografia, Instituto do Coracao (InCor), Hospital das Clinicas HCFMUSP, Faculdade de Medicina, Universidade de Sao Paulo, Sao Paulo, SP, BR; II Unidade de Reabilitacao Cardiovascular e Fisiologia do Exercicio, Instituto do Coracao (InCor), Hospital das Clinicas HCFMUSP, Faculdade de Medicina, Universidade de Sao Paulo, Sao Paulo, SP, BR; III Departamento de Biodin�mica do Movimento do Corpo Humano, Escola de Educação Física e Esporte, Universidade de Sao Paulo, Sao Paulo, SP, BR; IV Hospital Israelita Albert Einstein, São Paulo, SP, BR

**Keywords:** ECG Criteria, Left Ventricular Hypertrophy, Dynamic Exercise, Static Exercise, Cardiac Adaptation

## Abstract

**OBJECTIVES:**

In athletes, isolated electrocardiogram high voltage criteria are widely used to evaluate left ventricular hypertrophy, but positive findings are thought to represent normal electrocardiogram alterations. However, which electrocardiogram criterion can best detect left ventricular hypertrophy in athletes of various sport modalities remains unknown.

**METHODS:**

Five electrocardiogram criteria used to detect left ventricular hypertrophy were tested in 180 male athletes grouped according to their sport modality: 67% low-static and high-dynamic components and 33% high-static and high-dynamic components of exercise. The following echocardiogram parameters are the gold standard for diagnosing left ventricular hypertrophy: left ventricular mass index ≥134 g.m^-2^, relative wall thickness ≥0.42 mm, left ventricular diastolic diameter index ≥32 mm.m^-2^, septum wall thickness ≥13 mm, and posterior wall thickness ≥13 mm. Results for the various criteria were compared using the kappa coefficient. Significance was established at *p*<0.05.

**RESULTS:**

Fifty athletes (28%) presented with left ventricular hypertrophy according to electrocardiogram findings, with the following sensitivities and specificities, respectively: 38-53% and 79-83% (Perugia), 22-40% and 89-91% (Cornell), 24-29% and 90% (Romhilt-Estes), 68-87% and 20-23% (Sokolow-Lyon), and 0% and 99% (Gubner). The Perugia and Cornell criteria had higher negative predictive values for the low-static and high-dynamic subgroup. Kappa coefficients were higher for Romhilt-Estes, Cornell and Perugia criteria than for Sokolow-Lyon and Gubner criteria.

**CONCLUSION:**

All five evaluated criteria are inadequate for detecting left ventricular hypertrophy, but the Perugia, Cornell and Romhilt-Estes criteria are useful for excluding its presence. The Perugia and Cornell criteria were more effective at excluding left ventricular hypertrophy in athletes involved in a sport modality with low-static and high-dynamic component predominance.

## INTRODUCTION

The initial publications on the correlation of electrocardiogram (ECG) results for left ventricular hypertrophy (LVH), which were mostly based on voltage criteria (e.g., Sokolow-Lyon, Cornell, Gubner and many others), have shown the very low sensitivity and high specificity of ECG 1-5. However, none of these criteria were developed to evaluate an athlete’s heart, despite knowledge regarding the relevant heart adaptations caused by high intensity exercise training 6-10. This physiologic cardiac remodeling is related to chamber enlargement and increased volume and wall thickness, leading to an augmented left ventricular (LV) mass with normal systolic and diastolic functions 10. Moreover, the sport modality appears to influence cardiac remodeling and consequently the degree of LVH; these factors depend on the combination of the intensity (low, medium or high) of both the dynamic and static components of exercise 8,10-12. Interestingly, some resting ECG features, such as sinus bradycardia, first and second (Mobitz I) degree atrioventricular blocks, early repolarization, and isolated high QRS voltages, that are usually found in both amateur and professional athletes, were reported in some studies 8, 11. Many ECGs that fulfill the Sokolow-Lyon voltage criteria for LVH in trained athletes have been reported 8,13-16. Although the previous finding by Pelliccia et al. 8 verified that there were 20% more abnormal ECGs when using the criterion of an isolated QRS voltage increase, Calore et al. 14 suggested that positive results for an isolated QRS voltage increase should not be used in highly trained athletes when evaluating LVH. Accordingly, the Sokolow-Lyon criterion (QRS voltage analysis) seems to be inadequate for young competitive athletes 15. Singla et al. 16 confirmed that a high isolated QRS amplitude is a physiological exercise training response, rather than a pathological risk factor, as was postulated for athletes 11. Nevertheless, there is no clear information regarding the usefulness of other ECG criteria or regarding which criteria is the most appropriate for detecting LVH in athletes. In addition, whether the cardiac adaptation provoked by different sport modalities 12 influences the ECG-based detection of LVH in athletes is unknown. Accordingly, the present study attempts to determine which of five ECG criteria for LVH based on echocardiographic parameters best applies to athletes depending on their sport modality.

## METHODS

Population and Study Protocol *-* This cross-sectional retrospective study was conducted in 180 healthy professional and amateur male endurance athletes (76 soccer players, 44 long distance runners, 11 road cyclists, 25 rowers, 8 triathletes and 16 boxers; 15 to 60 years of age) who were engaged in competitive training and were listed in the database of the sport and exercise outpatient facility of a tertiary hospital. According to the Task Force 8: Classification of Sports 12, our study group was divided into two subgroups based on their components of exercise: low-static and high-dynamic (LSHD – 66.7%) and high-static and high-dynamic (HSHD – 33.3%) components of exercise. The present study was approved by the Institutional Ethics Committee for the Analysis of Research Projects (#0101/09) in accordance with the rules of this ethics committee and our country, with no need for individual signed consent forms.

Structural and Functional Cardiac Evaluations *-* Echocardiography is routinely performed to evaluate all athletes who are enrolled in the sport and exercise outpatient facility. Two-dimensional and Doppler echocardiographic studies were performed to assess the morphology of the left ventricle (LV) using a cardiac ultrasound machine (HP/Philips Sonos 5500 – Davis Medical Electronics Inc., The Netherlands). LV cavity diameters were obtained using M-mode with 2-dimensional guidance according to the guidelines of the American Society of Echocardiography 17. The LV mass was calculated as 0.8[1.04(LVDD + PWT + SWT)^3^ – LVDD^3^] + 0.6, with values provided in grams; (LVDD = left ventricular diastolic diameter; PWT = posterior wall thickness; SWT = septum wall thickness). The volumes were measured according to the modified Simpson’s rule, and the ejection fraction was calculated as (EDV - ESV) / EDV (EDV = end-diastolic volume; ESV = end-systolic volume). Relative wall thickness (RWT) was calculated as (2 x PWT) /LVDD. All of the echocardiographic analyses were performed with the investigator blinded to the ECG information.

The following criteria, considered the gold standards, were used to identify left ventricular hypertrophy (LVH) in the athletes 9,17-19. The results of this examination were accepted as a positive identification of LVH for the following conditions:

- Left ventricular mass index (LVMI) ≥134 g.m^-2^
- Relative wall thickness (RWT) ≥0.42 mm
- Left ventricular diastolic diameter index (LVDDI) ≥32 mm.m^-2^
- Septum wall thickness (SWT) ≥13 mm and/or
- Posterior wall thickness (PWT) ≥13 mm

The ECG criteria were tested against echocardiographic parameters for the following four subgroups: (1) LVMI; (2) RWT; (3) LVDDI and/or SWT and/or PWT; (4) LVMI or RWT or LVDDI and/or SWT and/or PWT.

12-Lead Resting Electrocardiogram *-* All athletes underwent 12-lead ECG examination after five minutes of resting. The ECG and echocardiogram of each athlete were obtained within the range of six months, a period in which the athletes maintained an exercise training regimen. Tracings were taken with a Philips PageWriter Trim II electrocardiograph (Philips Medical Systems, Andover, MA, USA) at 25 mm/sec after the proper calibration for an amplitude of 1 mV/cm. The usual ECG parameters (heart rate, Pr interval, QRS duration, QT and QTc intervals, and P, QRS and T axes) were analyzed.

The following five criteria that are most commonly used by the Hospital Electrocardiology Unit were chosen to identify LVH:

1. Sokolow-Lyon (2):
  R wave _(V1/V2)_ + S wave_ (V5/V6)_ ≥35 mm
2. Romhilt-Estes score (≥5 points) (4):
  3 points:
  - Frontal plane: R or S waves ≥20 mm or
  - Horizontal plane: R or S waves ≥30 mm
  - Morris index (left atrial enlargement in lead V1)
  - Strain pattern (ST segment deviation and negative T wave in lead V6)
  2 points:
  - Left axis deviation
  1 point:
  - QRS duration ≥100 ms
  - Intrinsicoid deflection

Although the 4-point score in the Romhilt-Estes analysis implies the possible presence of LVH, only one athlete among 17 was diagnosed with LVH according to echocardiographic criteria. Therefore, for our study, we decided to consider the 4-point score as a negative result for LVH.

3. Cornell (3):
  R wave _(aVL)_ + S wave_ (V3)_ ≥28 mm
4. Gubner (1):
  R wave _(DI)_ + S wave_ (DIII)_ ≥22 mm
5. Perugia (5):
  R wave_ (aVL)_ + S wave _(V3)_ ≥24 mm or strain pattern or ≥5 points for the Romhilt-Estes score

Anthropometric and Maximal Cardiopulmonary Exercise Capacity Evaluations *-* Height and body mass were measured to the nearest 0.1 cm and 0.1 kg, respectively. Body mass index was determined as weight (kg) divided by height squared (m^2^). Body surface area was obtained using the Dubois and Dubois formula 20. The individuals performed a progressive cardiopulmonary exercise test (93% and 33% of the athletes in LSHD and HSHD subgroups, respectively) on their specific ergometers (Treadmill: Inbramed Millennium ATL, Inbrasport, Porto Alegre, RS, Brazil; Cycle: Ergoline GmbH, ViaSprint 150P Analog, Palm Springs, CA, EUA; and Indoor rowing ergometer: Concept 2 PM4, model D, Vermont, USA) using a ramp protocol. The test duration ranged 8 to 17 minutes, as previously recommended 21. The oxygen and carbon dioxide outputs were measured via breath-by-breath analysis (Vmax SERIES 229, SensorMedics Corporation, California, USA) as described in a previous study 22.

### Statistical Analyses

Data for all variables are presented as the mean ± SD. The results using ECG criteria to diagnose LVH were compared to the results obtained by echocardiography. Continuous variables were compared using the non-paired T test, and categorical variables were analyzed using Fisher’s exact test (sensitivity, specificity, positive predictive value - PPV, and negative predictive value - NPV). The kappa coefficient was used to verify the agreement between each type of ECG criteria and the gold standard echocardiographic parameters. Kappa values were classified as <0 - Poor; 0–0.20 - Slight; 0.21–0.40 - Fair; 0.41–0.60 - Moderate; 0.61–0.80 - Substantial; and 0.81–1.00 - Almost perfect 23. Statistical analyses were performed using SPSS software. A *p* value ≤0.05 was considered significant.

## RESULTS

The characteristics and echocardiographic and ECG parameters of the athletes are shown in Table 1. The athletes had normal weights (BMI from 18.5 – 24.9 kg.m^-2^), and the peak VO_2_ values indicated that athletes had a high cardiopulmonary capacity. The HSHD subgroup had a higher peak VO_2_. Most athletes presented with LVH echocardiographic criteria within the normal range for this population. However, in 50 athletes (27.8%), at least one of the five measurements (LVMI, RWT, LVDDI, SWT, or PWT) was higher than the cutoff value for LVH, and the number of elevated values was equally distributed between the LSHD and HSHD subgroups. As a result of the predominance of the static exercise component in the HSHD subgroup, LVDDI was lower and RWT, SWT and PWT were higher in the HSHD subgroup than in the LSHD subgroup. The ECG findings were within the normal range for an adult male population. Each ECG criterion for LVH was applied, and the results were later compared to the echocardiogram results. The sensitivity, specificity, PPV, NPV and kappa coefficients are presented in Tables 2 to 5.

Left Ventricular Mass Index *vs.* ECG Criteria *–* The Perugia criterion was the only criteria that provided significant accuracy for correctly identifying LVH when using LVMI for the total cohort, as well as for the LSHD subgroup, in which the criteria presented an elevated negative predictive value. Despite this result, the kappa coefficient was considered fair. None of the other criteria showed any significant results, but they did exhibit lower kappa values than that of the Perugia criterion (Table 2).

Relative Wall Thickness *vs.* ECG Criteria *–* The Romhilt-Estes and Perugia criteria provided significant accuracy for correctly identifying LVH when using RWT for all athletes, and each of these criteria presented an elevated negative predictive value. The sport modality subgroups were not significantly different. Regardless of the accuracy, the kappa coefficient was considered fair for the Romhilt-Estes criterion and slight for the Perugia criterion. None of the other criteria provided any significant results other than low kappa values (Table 3).

Left Ventricular Diastolic Diameter Index and/or Septum and/or Posterior Wall Thickness *vs.* ECG Criteria *-* The Cornell and Perugia criteria provided significant accuracy for correctly identifying LVH when using LVDDI and/or Septum and/or PWT for all of the athletes and for the LSHD subgroup. Both criteria presented an elevated negative predictive value. Despite the accuracy, the kappa coefficient was considered fair for the Cornell criteria and slight for the Perugia criterion. None of the other criteria had any significant results other than low kappa values (Table 4).

Left Ventricular Mass Index or Relative Wall Thickness or Left Ventricular Diastolic Diameter Index and/or Septum and/or Posterior Wall Thickness *vs.* ECG Criteria *-* When using at least one of the three above-mentioned echocardiographic criteria together, the Cornell, Romhilt-Estes and Perugia criteria provided significant accuracy for correctly identifying LVH for all of the athletes. The sport modality subgroups did not show any significant results. The three criteria presented elevated negative predictive values, and the kappa coefficient was considered fair for the Perugia criterion and slight for the Cornell and Romhilt-Estes criteria. None of the other criteria presented any significant results other than low kappa values (Table 5).

## DISCUSSION

The present study highlights the poor accuracy of the five tested ECG criteria in correctly identifying LVH based on the results of the gold standard echocardiogram method in a population of athletes. The Perugia, Cornell and Romhilt-Estes ECG criteria presented high negative predictive values, which could be helpful for excluding the presence of LVH in athletes. Specifically, the Perugia and Cornell criteria were more effective for excluding LVH in athletes involved in sport modalities with a predominance of the dynamic component. We understand that concentric and eccentric physiological cardiac adaptations occur in athletes involved in sport modalities with this characteristic. Thus, the pathological cardiac increase would involve additional electrocardiographic changes in addition to the high voltage changes.

In our study, physiologic LVH was identified using at least one of the echocardiographic criteria in 50 athletes of the total cohort (27.7%). When considering each ECG criterion alone, the Perugia criterion was the one that came closest to the echocardiographic LVH diagnosis (23.3%), followed by the Romhilt-Estes score (13.9%) and the Cornell criterion (13.3%). The approximation for the Perugia criterion may have been influenced by both the Cornell and Romhilt-Estes criteria. In contrast, the Sokolow-Lyon criterion overestimated (77.8%) the presence of LVH, and the Gubner criterion underestimated it (0.6%). Of the three ECG criteria that presented significant results for sensitivity and specificity, the Perugia criterion showed a higher sensitivity (38-53%), followed by the Cornell criterion (22-40%) and the Romhilt-Estes score (24-29%). Notably, the sensitivity of the Perugia-based identification increased when the LVMI and LVDDI and/or septum and/or PWT echocardiographic criteria were used. Although Schillaci et al. 24 (hypertensive and older individuals) evaluated a different population, their Perugia ECG criterion sensitivity was comparable to that of our study (39%). Our sensitivity results for the Romhilt-Estes and Cornell criteria were similar to that of Gasperin et al. (35% and 38%, respectively), who studied healthy individuals 25. In the original publication 4, the sensitivity for the Romhilt-Estes criterion was much higher (60%) than in our present study. However, this difference is probably due to the population they studied, which included individuals with serious cardiac disease. These results confirm that none of the studied ECG criteria were valid for identifying LVH in athletes. In addition, in our study, all of the criteria provided similar specificity results: the Perugia (79-83%), Cornell (89-91%) and Romhilt-Estes (90%) specificities were comparable to the results in healthy individuals 25 but were slightly lower than those for a population with heart disease 4,24.

Subgroup analyses showed that the Perugia (Tables 2 and 4) and Cornell (Table 4) criteria could provide important additional information for the LSHD subgroup. The sensitivity was 46-55% and 36%, and the specificity was 86% and 92% (Perugia and Cornell, respectively). These two ECG criteria could be useful for excluding the presence of LVH in athletes involved in sport modalities with low-static and high-dynamic components of exercise, such as long distance running and soccer.

All diagnoses of LVH were equally distributed in the LSHD and HSHD subgroups (25 athletes each). LVH was found in 9% and 20% of LSHD and HSHD, respectively, when the LVMI echocardiographic criterion was applied. When RWT was used, LVH was present in 10% and 37% of LSHD and HSHD, respectively. Finally, the LVDDI and/or septum and/or PWT criteria revealed the presence of LVH in 9% and 7% of athletes in the LSHD and HSHD subgroups, respectively. These results clearly demonstrate the influence of sport modality on structural cardiac adaptations. As expected, the predominance of the dynamic component leads to hypertrophy, mainly due to cavity increase (dilatation); furthermore, the predominance of the static component leads to hypertrophy, mainly due to a thickness increase 12.

Despite the poor positive predictive value, the Perugia criterion could be used to identify athletes without LVH in 78% to 95% of the study population, according to all echocardiographic criteria. Similarly, the Romhilt-Estes and Cornell criteria were useful for identifying athletes without LVH in 75% to 84% and 75% to 94%, respectively, of the study population, with the use of only two echocardiographic criteria. Neither the Sokolow-Lyon nor the Gubner criteria should be used to evaluate this population, because these criteria could not identify or exclude LVH in athletes. Thus, our results demonstrate that the Sokolow-Lyon criterion should be used with caution, as previously demonstrated 14, and any ECG alteration identified by this set of criteria might mainly be due to physiological adaptations related to exercise training 16. The sport modality subgroup analyses showed higher negative predictive values for the Perugia and Cornell criteria in the LSHD subgroup, mainly when LVDDI and/or septum and/or PWT echocardiographic criteria were used. This finding could have a practical implication, since this subgroup of athletes (LSHD) presented with higher LVDDI and lower septum and PWT than did the HSHD subgroup. Thus, for the athletes involved in sports with a predominance of the dynamic component, the negative predictive value for the Perugia and Cornell criteria was higher. This finding implies an almost 100% correct exclusion of cardiac disease (LVH) when those criteria are not met in these athletes.

The kappa coefficients obtained for all ECG criteria showed that the results based on the Sokolow-Lyon (-0.06 to 0.02) and Gubner (-0.01) criteria were definitely obtained by chance. In contrast, the kappa coefficients for the Romhilt-Estes (0.10 to 0.21), Cornell (0.07 to 0.23) and Perugia (0.18 to 0.23) criteria were higher than those for the Sokolow-Lyon and Gubner criteria, albeit the values were low. There are two possible explanations for the low (fair) values of the kappa coefficient for these three criteria. First, those ECG criteria are not useful for identifying LVH. Second, the low LVH prevalence in our population (27.7%) led to low reproducibility.

In conclusion, the Perugia, Cornell and Romhilt-Estes ECG criteria appear to be the most appropriate criteria with which to exclude the presence of LVH in athletes. Additionally, using the Perugia and Cornell criteria improved the exclusion of LVH for the athletes involved in a sport modality with a predominance of the low-static and high dynamic components.

### Study Limitations

We recognize that some limitations exist for the present study. First, the use of male athletes limited the results and conclusions to this gender. Second, a higher number of athletes with LVH would be desirable. However, this condition would limit the sample size because of the low incidence of LVH in athletes (27.7% in our study when considering several echocardiographic parameters). Furthermore, Pelliccia et al. 19 reported that only 15% of elite athletes had a left ventricular cavity ≥60 mm.

### AUTHOR CONTRIBUTIONS

Samesina N and Azevedo LF provided substantial contributions to research design, or the acquisition, analysis or interpretation of data. Samesina N, Azevedo LF and de Matos LD were responsible for the manuscript draft and critical revision. All the authors submitted and approved the final version of the manuscript.

## Figures and Tables

**Table 1:** Baseline Parameters.

	Total (180)	LSHD (120)	HSHD (60)	*p*
	Baseline Characteristic			
Age (years)	26±9	26±10	28±7	0.20
Body mass (kg)	71.8±9.5	70.9±9.5	73.7±9.5	0.06
Height (m)	1.77±0.08	1.76±0.08	1.78±0.08	0.23
BMI (kg.m^-2^)	22.7±1.9	22.7±1.9	23.3±2.2	0.10
BSA (m^2^)	1.88±0.16	1.87±0.16	1.91±0.15	0.09
Peak VO_2_ (L.min^-1^)	4.24±0.80	4.04±0.66	5.39±0.43	<0.001
	Echo			
LVMI (g.m^-2^)	106.2±19.2	105.2±18.6	108.1±20.2	0.346
RWT (cm)	0.37±0.05	0.36±0.04	0.39±0.05	<0.000
LVDDI (mm.m^-2^)	28.1±2.4	28.5±2.2	27.4±2.5	0.003
SWT (cm)	1.00±0.11	0.98±0.11	1.04±0.11	0.001
PWT (cm)	0.96±0.11	0.94±0.10	1.01±0.11	<0.000
LVEF (%)	0.64±0.06	0.64±0.06	0.63±0.06	0.112
	ECG			
HR (bpm)	54±10	53±8	57±12	0.01
Pr (ms)	166±25	167±26	165±22	0.74
QRS (ms)	92±9	92±9	94±8	0.10
QT (ms)	420±41	422±43	414±33	0.31
QTc (ms^-1^)	393±30	393±31	395±29	0.70
P (^o^)	46±27	46±28	47±23	0.81
QRS (^o^)	63±41	63±40	63±42	0.99
T (^o^)	33±27	30±20	42±40	0.04

**BMI** body mass index; **BSA** body surface area; **HR** heart rate; **HSHD** high-static and high-dynamic; **LSHD** low-static and high-dynamic; **LVDDI** left ventricular diastolic diameter index; **LVEF** left ventricular ejection fraction; **LVMI** left ventricular mass index; **PWT** posterior wall thickness; **QTc** corrected QT interval; **RWT** relative wall thickness; **SWT** septum wall thickness.

**Table 2:** LVMI x Electrocardiogram Criteria.

	Total (179)			LSHD (119)			HSHD (60)		
	Estimate	CI (95%)		Estimate	CI (95%)		Estimate	CI (95%)	
		Lower	Upper		Lower	Upper		Lower	Upper
Sokolow-Lyon									
Sensitivity	82.6	61.2	95.1	100.0	71.5	100.0	66.7	34.9	90.1
Specificity	23.1	16.7	30.5	15.7	9.5	24.1	39.6	25.8	54.7
PPV	13.7	8.4	20.5	10.8	55.1	18.5	21.6	9.8	38.3
NPV	90.0	76.4	97.2	100.0	80.5	100.0	82.6	61.2	95.5
P	0.789			0.361			0.752		
Kappa Coefficient	0.018	-0.037	0.073	0.033	0.008	0.058	0.035	-0.134	0.204
Cornell									
Sensitivity	26.1	10.2	48.4	27.3	6.0	61.0	25.0	5.5	57.2
Specificity	89.1	83.1	93.5	91.7	84.8	96.1	83.3	69.8	92.5
PPV	26.1	10.2	48.4	25.0	5.5	57.2	27.3	6.0	61.0
NPV	89.1	83.1	93.5	92.5	85.8	96.7	81.6	68.0	91.2
P	0.086			0.082			0.677		
Kappa Coefficient	0.152	-0.030	0.334	0.182	-0.071	0.435	0.086	-0.188	0.360
Gubner									
Sensitivity	0	0	0.1	0	0	28.5	---	---	---
Specificity	99.4	96.5	100.0	99.1	95.0	100.0	---	---	---
PPV	0	0	97.5	0	0	97.5	---	---	---
NPV	87.1	81.3	91.6	96.7	83.9	95.3	---	---	---
P	1.000			1.000			---		
Kappa Coefficient	-0.011	-0.031	0.009	-0.016	-0.045	0.013	---	---	---
Romhilt-Estes									
Sensitivity	26.1	10.2	48.4	18.2	2.3	51.8	33.3	9.9	65.1
Specificity	87.8	81.6	92.5	93.5	87.1	98.0	75.0	60.4	86.3
PPV	24.0	9.3	45.1	22.2	2.8	60.0	25.0	7.3	52.4
NPV	89.0	82.9	93.4	91.8	85.0	96.2	81.8	67.3	91.8
P	0.101			0.195			0.716		
Kappa Coefficient	0.134	-0.044	0.312	0.127	-0.122	0.376	0.074	-0.187	0.335
Perugia									
Sensitivity	47.8	26.8	69.4	45.5	16.8	76.6	50.0	21.1	78.9
Specificity	80.8	73.7	86.6	86.1	78.1	92.0	68.8	53.7	81.4
PPV	26.8	14.2	42.9	25.0	8.6	49.1	28.6	11.3	52.2
NPV	91.3	85.3	95.4	93.9	87.3	97.7	84.6	69.5	94.1
P	0.006			0.019			0.312		
Kappa Coefficient	0.214	0.049	0.379	0.231	0.006	0.456	0.146	-0.101	0.393

**HSHD** high-static and high-dynamic; **LSHD** low-static and high-dynamic; **LVMI** left ventricular mass index; **NPV** negative predictive value; **PPV** positive predictive value.

**Table 3:** RWT x Electrocardiogram Criteria.

	Total (179)			LSHD (119)			HSHD (60)		
	Estimate	CI (95%)		Estimate	CI (95%)		Estimate	CI (95%)	
		Lower	Upper		Lower	Upper		Lower	Upper
Sokolow-Lyon									
Sensitivity	67.7	49.5	82.6	75.0	42.8	94.5	63.6	40.7	82.8
Specificiy	20.0	13.8	27.5	13.1	7.3	21.0	39.5	24.1	56.7
PPV	16.6	10.8	23.8	8.8	4.1	16.1	37.8	22.5	55.3
NPV	72.5	56.2	85.4	82.4	56.6	96.2	65.2	42.8	83.6
P	0.168			0.376			1.000		
Kapa Coefficint	-0.057	-0.137	0.023	-0.028	-0.089	0.033	0.027	-0.194	0.248
Cornell									
Sensitivity	17.7	6.8	34.6	8.3	0.2	38.5	22.7	7.8	45.4
Specificity	88.3	81.9	93.0	89.7	82.4	94.8	84.2	68.7	94.0
PPV	26.1	10.2	48.4	8.3	0.2	38.5	45.5	16.8	76.6
NPV	82.1	75.1	87.7	89.7	82.4	94.8	65.3	50.3	78.3
P	0.393			1.000			0.511		
Kappa Coefficient	0.068	-0.089	0.225	-0.019	-0.186	0.148	0.078	-0.155	0.311
Gubner									
Sensitivity	0	0	10.3	0	0	26.5	---	---	---
Specificity	99.3	96.2	100.0	99.1	94.9	100.0	---	---	---
PPV	0	0	97.5	0	0	97.5	---	---	---
NPV	80.9	74.4	86.4	89.8	82.9	94.6	---	---	---
P	1.000			1.000			---		
Kappa Coefficient	-0.011	-0.033	0.011	-0.016	-0.045	0.013	---	---	---
Romhilt-Estes									
Sensitivity	29.4	15.1	47.5	8.3	0.2	38.5	40.9	20.7	63.7
Specificity	89.7	83.5	94.1	92.5	85.8	96.7	81.6	65.7	92.3
PPV	40.0	21.1	61.3	11.1	0.2	48.3	56.3	29.9	80.2
NPV	84.4	77.8	89.8	90.0	82.8	94.9	70.5	54.8	83.3
P	0.010			1.000			0.074		
Kappa Coefficient	0.212	0.038	0.386	0.01	-0.174	0.194	0.239	-0.012	0.490
Perugia									
Sensitivity	38.2	22.2	56.4	16.7	2.1	48.4	50.0	28.2	71.8
Specificity	80.7	73.4	86.8	83.2	74.8	89.7	73.7	56.9	86.6
PPV	31.7	18.1	48.1	10.0	1.2	31.7	52.4	29.8	74.3
NPV	84.8	77.7	90.3	89.9	82.1	95.1	71.8	55.1	85.0
P	0.024			1.000			0.093		
Kappa Coefficient	0.175	0.012	0.338	-0.001	-0.173	0.171	0.239	-0.014	0.492

**HSHD** high-static and high-dynamic; **LSHD** low-static and high-dynamic; **NPV** negative predictive value; **PPV** positive predictive value; **RWT** relative wall thickness.

**Table 4:** LVDDI and/or Septum and/or PWT x Electrocardiogram Criteria.

	Total (179)			LSHD (119)			HSHD (60)		
	Estimate	CI (95%)		Estimate	CI (95%)		Estimate	CI (95%)	
		Lower	Upper		Lower	Upper		Lower	Upper
Sokolow-Lyon									
Sensitivity	86.7	59.6	98.3	90.9	58.7	99.8	75.0	19.4	99.4
Specificity	23.0	16.9	30.2	14.7	8.6	22.8	39.3	26.5	53.2
PPV	9.3	5.0	15.4	9.7	4.8	17.1	8.1	1.7	21.9
NPV	95.0	83.1	99.4	94.1	71.3	99.9	95.7	78.1	99.9
P	0.527			1.000			1.000		
Kappa Coefficient	0.02	-0.019	0.059	0.012	-0.027	0.051	0.03	-0.066	0.126
Cornell									
Sensitivity	40.0	16.3	67.7	36.4	10.9	69.2	50.0	6.8	93.2
Specificity	89.1	83.3	93.4	91.7	84.9	96.2	83.9	71.7	92.4
PPV	25.0	9.8	46.7	30.8	91.0	61.4	18.2	2.3	51.8
NPV	94.2	89.3	97.3	93.5	87.0	97.3	95.9	86.0	99.5
P	0.007			0.018			0.150		
Kappa Coefficient	0.229	0.029	0.429	0.26	-0.001	0.521	0.187	-0.111	0.485
Gubner									
Sensitivity	0	0	21.8	0	0	28.5	---	---	---
Specificity	99.4	96.7	100.0	99.1	95.0	100.0	---	---	---
PPV	0	0	97.5	0	0	97.5	---	---	---
NPV	91.6	86.6	95.2	90.8	84.1	95.3	---	---	---
P	1.000			1.000			---		
Kappa Coefficient	-0.011	-0.031	0.009	-0.016	-0.043	0.011	---	---	---
Romhilt-Estes									
Sensitivity	26.7	7.8	55.1	27.3	6.0	61.0	25.0	0.6	80.6
Specificity	87.3	81.2	91.9	94.5	88.4	98.0	73.2	59.7	84.2
PPV	16.0	4.5	36.1	33.3	7.5	70.1	6.3	0.2	30.2
NPV	92.9	87.7	96.4	92.8	86.3	96.8	93.2	81.4	98.6
P	0.232			0.036			1.000		
Kappa Coefficient	0.107	-0.069	0.283	0.237	-0.039	0.513	-0.007	-0.191	0.177
Perugia									
Sensitivity	53.3	26.6	78.7	54.6	23.4	83.3	50.0	6.8	93.2
Specificity	79.4	72.4	85.3	86.2	78.3	92.1	66.1	52.2	78.2
PPV	19.1	8.6	34.1	28.6	11.3	52.2	9.5	1.2	30.4
NPV	94.9	89.8	97.9	95.0	88.6	98.3	94.9	82.7	99.4
P	0.008			0.004			0.606		
Kappa Coefficient	0.18	0.025	0.335	0.29	0.065	0.515	0.054	-0.122	0.230

**HSHD** high-static and high-dynamic; **LSHD** low-static and high-dynamic; **LVDDI** left ventricular diastolic diameter index; **NPV** negative predictive value; **PPV** positive predictive value; **PWT** posterior wall thickness.

**Table 5:** LVMI or RWT or LVDDI and/or Septum and/or PWT x Electrocardiogram Criteria.

	Total (179)			LSHD (119)			HSHD (60)		
	Estimate	CI (95%)		Estimate	CI (95%)		Estimate	CI (95%)	
		Lower	Upper		Lower	Upper		Lower	Upper
Sokolow-Lyon									
Sensitivity	74.0	59.7	85.4	84.0	63.9	95.5	64.0	42.6	82.0
Specificity	20.9	14.3	29.0	13.8	7.6	22.5	40.0	23.9	57.9
PPV	26.2	19.5	34.8	20.6	13.2	29.7	43.2	27.1	60.5
NPV	67.5	50.9	81.4	76.5	50.1	93.2	60.9	38.6	80.3
P	0.549			0.754			0.794		
Kappa Coefficient	-0.030	-0.120	0.060	-0.008	-0.082	0.066	0.037	-0.196	0.270
Cornell									
Sensitivity	22.0	11.5	36.0	20.0	6.8	40.7	24.0	90.3	45.1
Specificity	90.7	84.3	95.1	92.6	85.3	97.0	85.7	69.8	95.2
PPV	47.8	26.8	69.4	41.7	15.2	72.3	54.6	23.4	83.3
NPV	75.0	67.5	81.6	81.3	72.7	88.2	61.2	46.2	74.8
P	0.043			0.126			0.500		
Kappa Coefficient	0.169	0.022	0.316	0.191	-0.011	0.394	0.106	-0.115	0.327
Gubner									
Sensitivity	0	0	7.1	0	0	13.7	---	---	---
Specificity	99.2	95.8	100.0	98.9	94.2	100.0	---	---	---
PPV	0	0	97.5	0	0	97.5	---	---	---
NPV	71.9	64.7	78.4	78.8	70.3	85.8	---	---	---
P	1.000			1.000			---		
Kappa Coefficient	-0.011	-0.033	0.011	-0.016	-0.047	0.015	---	---	---
Romhilt-Estes									
Sensitivity	24.0	13.1	38.1	12.0	2.5	31.2	36.0	18.0	57.5
Specificity	89.9	83.4	94.5	93.6	86.6	97.6	80.0	63.1	91.6
PPV	48.0	27.8	68.7	33.3	7.5	70.1	56.3	29.9	80.2
NPV	75.3	67.7	81.9	80.0	71.3	87.0	63.4	47.8	77.6
P	0.028			0.395			0.238		
Kappa Coefficient	0.159	0.010	0.308	0.068	-0.104	0.240	0.169	-0.072	0.410
Perugia									
Sensitivity	38.0	24.6	52.9	28.0	12.1	49.4	48.0	27.8	68.7
Specificity	83.0	75.3	89.0	86.2	77.5	92.4	74.3	56.8	87.5
PPV	46.3	30.6	62.6	35.0	15.4	59.3	57.1	34.1	78.2
NPV	77.5	69.7	84.2	81.8	72.8	88.8	66.7	49.8	80.9
P	0.005			0.129			0.102		
Kappa Coefficient	0.234	0.079	0.389	0.182	-0.020	0.384	0.228	-0.021	0.477

**HSHD** high-static and high-dynamic; **LSHD** low-static and high-dynamic; **LVDDI** left ventricular diastolic diameter index; **LVMI** left ventricular mass index; **NPV** negative predictive value; **PPV** positive predictive value; **PWT** posterior wall thickness; **RWT** relative wall thickness.
